# Associating liver partition and portal vein ligation (ALPPS): A two staged procedure, in Bogotá Colombia. Case report and literature review

**DOI:** 10.1016/j.ijscr.2021.106560

**Published:** 2021-11-18

**Authors:** Carlos Eduardo Rey Chaves, Danny Conde, Laura Tapias, Isabella Roa, Juan Carlos Sabogal Olarte

**Affiliations:** a Hospital Universitario Mayor Méderi, Colombia; b School of Medicine, Universidad el Rosario, Colombia

**Keywords:** ALPPS, Liver, Technique, Postoperative liver failure, Liver partition, Liver tumors

## Abstract

**Introduction:**

For liver tumors (primary or metastases), surgery combined with neoadjuvant, or adjuvant chemotherapy is the treatment of choice, offering long term survival time and disease-free time period (Alvarez et al., 2012) Associating liver partition and portal vein ligation, or ALPPS, it's a surgical technique that increases the future liver remnant in a short period of time, trying to avoid postoperative liver failure (PLF), and achieving R0 resections in liver malignant tumors (Alvarez et al., 2012).

**Presentation of the case:**

A 43 years old woman with colorectal liver metastases in both lobes. Colorectal surgical procedure was performed 1 year previous the liver intervention, followed by adjuvant chemotherapy. Decision of a tri-segmental hepatectomy was made to resolve the metastases. Into the surgical procedure, we evaluated the liver parenchyma, and the future liver remnant tissue was insufficient, for that reason we decided to perform ALPPS procedure.

**Discussion:**

Colorectal liver metastases (CLRM) are considered the most common indication for ALPPS procedure according to the international registry. Compared with the portal vein ligation, resection rate varies from 50 to 80%, and the non-resectability disease was explained by tumor progression. Postoperative mortality rate was 5.1% in young patients (<60 years old), and 8% in general for CRLM. Oncologic outcomes represent an increased disease-free survival period and overall survival time compared with non-surgical approach.

**Conclusion:**

The ALPPS procedure it's an interesting approach to patients with not enough liver remnant tissue, with good oncologic results in terms of disease-free survival time, and overall survival. Appropriate selection of the patient, careful postoperative management, and a multidisciplinary approach are related with good postoperative outcomes.

## Highlights

• ALPPS should be in the surgical armamentarium in liver metastases from colorectal neoplasms, as well hepatocarcinoma and cholangiocarcinoma.
• Multidisciplinary approach in liver metastases impact in postoperative outcomes
• Adequate selection of patient reflects in postoperative outcomes and prolonged disease-free survival and overall survival period.
• This case report, it’s the first one published in Colombia.

## 1 Introduction and importance

Associating liver partition and portal vein ligation for staged hepatectomy, or ALPPS procedure, is a surgical strategy for patients with “unresectable” liver tumors, primary (as liver carcinoma) or secondary (colorectal liver metastases) [1]. Advances in anaesthesia, perioperative, and postoperative care, surgical devices, and techniques, have improved the outcomes in major liver resections, for that reason, the resection criteria are changing through the years. In the present time, hepatobiliary surgeons should focus on the remnant liver tissue in the postoperative time, and not in the number or localization of the tumors [2], [3].

This procedure helps to avoid a feared and severe complication in major or extended liver resection: Post-hepatectomy liver failure (PHLF). In patients with normal liver function, and no chronic liver illness, a FLRV of 25% of total liver volume it's enough to maintain the liver function in the postoperative time. In other patients, with chronic liver illness, or abnormal liver function due to toxics (systemic chemotherapy, radiation, sorafenib, etc.), we have to achieve near 35–40% of future liver remnant volume (FLRV) of total liver volume to avoid liver failure [3].

Post-hepatectomy liver failure, it's a limiting factor in liver resections. For that reason, rising techniques and strategies have the target in preventing this complication. In the 1980s, Makuuchi et al. reported the portal vein embolization, trying to generate contralateral hypertrophy of the hepatic lobe [4], [5], [6].

In 2007, a new surgical technique has been reported by Schnitzbauer et al.; that consists in the partition of the liver parenchyma with portal vein ligation; after that, Santibañez proposes a new name; “Associating liver partition with portal vein ligation for staged hepatectomy, or ALPPS” [7], [8].

Surgical technique consists in the transection of the liver parenchyma, with the ligation of the portal vein in a one or two stage hepatectomy. This procedure has the principal aim to increase the future liver remnant volume (FLRV), ALPPS increase approximately 20% of entire liver tissue, and increase up to 80% of the future liver remnant volume [9].

## 2 Presentation of the case

**Fig. 1:**
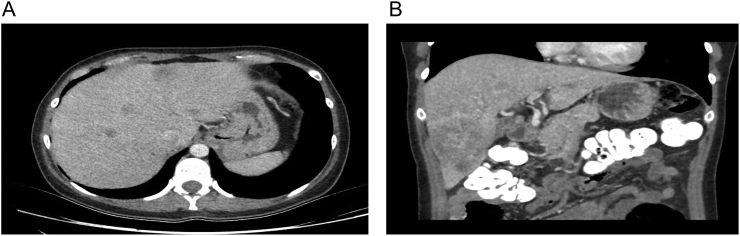
A: Liver metastases involving right and left hepatic lobes (segments 8, 4, 2, 3). B: Major liver tumor localized in segment 6 and 7 – Right hepatic lobe.

After ethical and institutional approval, previous informed consent filled, following SCARE guidelines [10]. Our paper present a 43-year-old woman presented initially with sigmoid colon primary tumor (adenocarcinoma, moderate differentiation; nodal status (25/60 nodes resected), TNM: T3N2M1), with liver metastases that compromises 5 and 6 anatomical segments of the liver (Fig. 1A). The major tumor localized in the right lobe of 85 mm (Fig. 1B). Multidisciplinary board was performed, with the decision of resection of colorectal mass (laparoscopic sigmoid resection) first followed by adjuvant chemotherapy. After almost 1 year of chemotherapy, re-stadification of the oncological status was made with a new abdominal MRI, that shows no change of the liver metastases. For that reason, the decision of a tri-segmental hepatectomy was made to resolve the metastases by a hepatobiliary and pancreatic surgeon.

After 4 weeks after the last cycle of chemotherapy, the patient underwent the surgical procedure. We evaluated the liver parenchyma with intraoperative echography, that shows metastases in liver segments 2, 3, 6, 7, and 8. We considered that FLRV was insufficient; for that reason we decided to perform an ALPPS procedure.

**Fig. 2:**
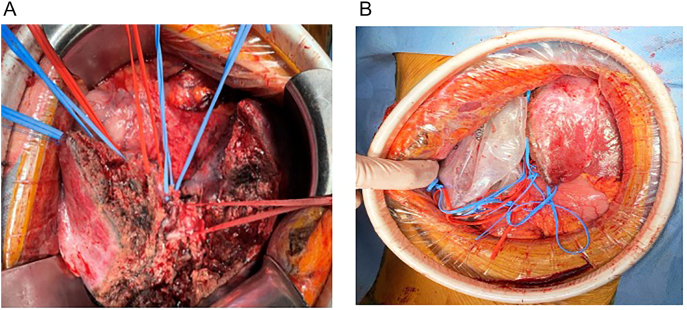
a: Vessel loops in the dissected portal triad. b: Exclusion of right hepatic lobe with a Bogota Bag.

First stage surgery was performed with the ligation of the right portal vein, and resection of the left lobe metastases (two-segment hepatectomy) with bipolar energy. Due to the involvement of 4 segment of the liver, we perform a cholecystectomy for the risk of malignant disease. Using vessel loops, we carefully dissect the portal triad, and exclude the right and left hepatic artery, portal vein, and also the biliary tract (Fig. 2a). We don't perform an abdominal closure, and exclude the liver with a Bogotá Bag using a sterile 3 liter saline solution bag (Fig. 2b).

## 3 Results

Operative time in the first and second stage of the surgery was 360 min, and 120 min, respectively. Estimated blood loss was 800 mL and 300 mL. Patients need in the first stage of ALPPS, the transfusion of 2 units of red blood cells. Intraoperative, require inotropic support with 0.08 mg/kg/min of noradrenaline. After the first stage, patients need an ICU stay of 2 days because of hyperlactatemia. We use ringer lactate as hidric support, and bolus of N-acetyl-cysteine every 8 h to prevent liver failure all 7 days after the tomographic control. Also we control magnesium, phosphate, and phosphorus with nutritional support. With no need to replace intravenous components. Liver enzymes were elevated to a maximum of 3200 and 2900 (AST–ALT respectively), after day 3 of first stage, enzymes reached a normal point.

**Fig. 3:**
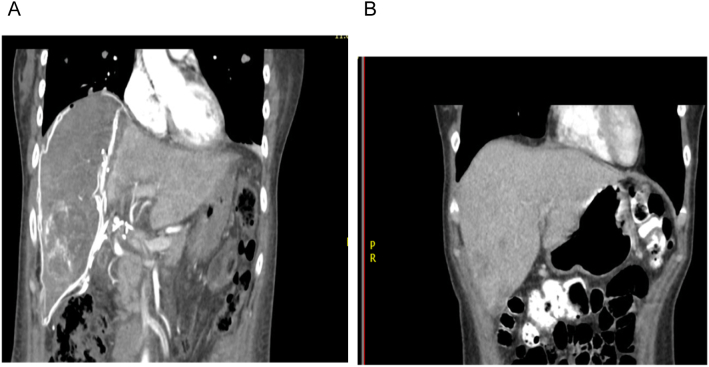
A: 7 day postoperative abdominal CT. Left lobe of the liver with 51% of hypertrophy (coronal view). B: Pre-operative abdominal CT. Comparison of the left lobe (coronal view).

**Fig. 4:**
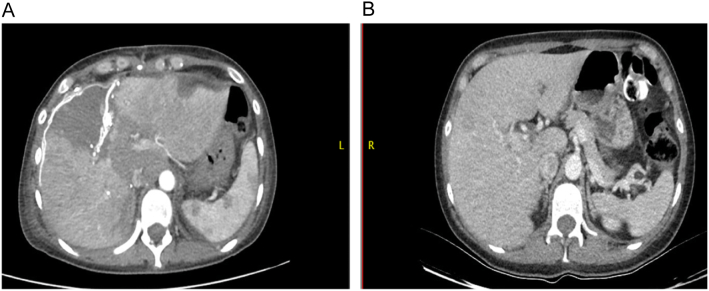
A: 7 day postoperative abdominal CT. Left lobe of the liver with 51% of hypertrophy (sagittal view). B: Pre-operative abdominal CT. Comparison of the left lobe (sagittal view).

After 7 days of postoperative management, we perform an abdominal CT with triple-contrast to check the remnant future liver volume. The left lobe grows approximately 51% percent compared with previous images (514 cm^3^ vs 1120 cm^3^) (Fig. 3, Fig. 4). The calculated FLRV after the left hypertrophy was 41% versus 21% in the first liver valoration.

**Fig. 5:**
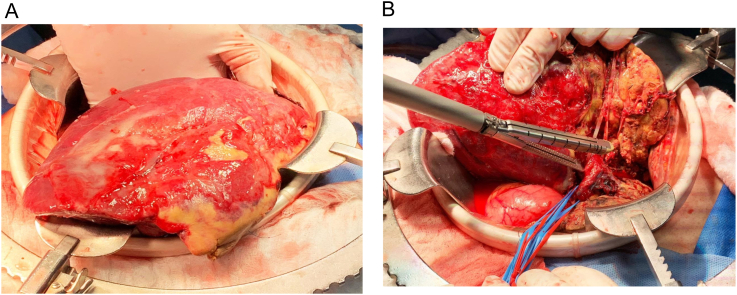
A: Right hepatic lobe. Evidence of necrotic tissue and inflammatory changes due to controlled ischemia. B: Ligation with mechanical suture of the right portal vein, end of hepatectomy.

Second stage surgery was performed finalizing the right hepatectomy, both stages with an open approach (Fig. 5). In this procedure, patients do not need any inotropic or transfusional support. We evaluate the perfusion of the left lobe with adequate flow of hepatic artery and left portal vein. Need 3 days of general hospitalization stay, and a total length of hospitalization of 13 days. With no postoperative complications. After the second stage, we don't see liver enzyme alteration. As well, bilirubin levels and coagulation times were normal all the postoperative time.

Pathology reports compromise of the gallbladder neck with adenocarcinoma and tumoral lesion of 5 cm in the 4 segments of the liver. In the right hepatic lobe, pathology shows a 9 × 7 centimeter tumor (adenocarcinoma), with a tumoral viability of 10% with a grade 2 of tumoral regression after chemotherapy. Resection borders are negative in all the pathology pieces.

After 30 days after hospital discharge, patients do not have any complications. Blake drainage was retired at 15 postoperative days. 6 months follow up do not show tumoral relapse, or any complication after surgery. Tumoral biomarkers (Ca 19-9, CEA) have negative results. Patient recovery after 3 months was successful, with recovery of the weight, and functional status. Genetic status of the patients was analyzed by MYRISK gen analysis, with negative mutations for hereditary tumors.

## 4 Discussion

Liver resections are in most of the cases the only curative treatment for primary lesions. Through the evolution of surgery, hepatic resections are considered suitable for metastatic disease, with increasing literature that report oncological benefits [10]. Important facts in liver procedures has been described, the most important one is the resectability depending on the FLRV, for that reason evolution in hepatic surgery were focused on surgical techniques that could improve the remnant tissue, and avoid postoperative liver failure; with positive oncologic outcomes (R0 resections, overall survival, and disease free survival time) [10], [11]. For that reason, novel procedures such as portal vein ligation, and embolization appears with impact in R0 resections; against these interventions was the increased risk of tumoral progression; for that reason, a two staged hepatectomy was described in 2007; with less time to achieve liver hypertrophy; reaching R0 resections in previous unresectable disease [10], [11].

In 2012 a case series of 25 patients that underwent ALPPS procedure was published, with acceptable morbidity and mortality rates, with zero rates of postoperative liver failure. For that reason an ALPPS registry was created in order to explore the novel technique, data in 2015 report a total of 583 cases performed.

These novel procedure show important benefits decreasing the risk of postoperative liver failure after a major liver resection; multiple interventions such as portal vein ligation or embolization were described, however, ALPPS procedures shows a fast, and safe hypertrophy (30 mL per day) [11], [12], with less proportion of liver failure after the surgery (8% in large series of cases) [12].

Not only the risk reduction of PLF, but the impact in increasing R0 resection has been described with these techniques. Increased number of lesions that involves a large proportion of the parenchyma lead to define a patient with unresectable disease; the possibility to offer the patient new liver tissue in a short period of time impact directly in the free margin resection with positive oncologic outcomes [5]. Other interventions as portal vein ligation, increase the risk of tumoral progression, or procedure failure; in contrast, the ALPPS procedure gives the possibility to the surgeon to perform liver resection in a reduced time, avoiding the risk of achieving oncologic resections [5], [11], [13].

Colorectal liver metastases (CLRM) are considered the most common indication for ALPPS procedure according to the international registry, although, could be an appropriate surgical approach in other cases of primary liver tumors, such as hepatocellular carcinoma and cholangiocarcinoma [12]. Compared with the portal vein ligation, resection rate varies from 50 to 80%, and the non-resectability disease was explained by tumor progression [14]. Initial oncologic impact in these patients, are described by Tanaka et al., with the important reduction of ki67 expression after the second stage hepatectomy [15]. In recent literature, an increased resection rate for colorectal liver metastases with ALPPS was reported with 97,1% [16]. Postoperative complications should be considered in any surgical technique; initially, for ALPPS, mortality rate was 5.1% in young patients (<60 years old), and 8% in general for CRLM [12]. However, experienced centers don't report 30 days postoperative mortality as Hernandez et al. shows [17]. In our case, we do not have any postoperative complications after 30–60-90 days.

Disease free survival time (DFS) and overall survival (OS) are also important matters in oncologic resections; according to the international ALPPS registry, DFS at 1 year was 59% and 41% in 2 years analyzed [12]. In terms of OS, after 6 months it was 86%, which decreased after 2 years of treatment to 59% [12]. Risk factors for early tumoral relapse has been described, the presence of >4 metastases could be the most important one, showing similar outcomes compared with portal vein ligation [11].

In order to achieve positive oncologic outcomes, Hernandez et al. describe the importance of a good selection criteria for patients that underwent ALPPS: 1. No evidence of extrahepatic disease, 2. good functional capacity, 3. complete or partial response to systemic treatment after 6 cycles [17]; these criteria show an OS of 100% after 9 months, and a mean DFS of 9.4 months. These criteria were adjusted to our patient, and at the time of the follow up (7 months), no evidence of tumoral relapse (clinical, biochemical, and tomographic) was found, with any complication after 7 months of surgery.

To the present time, this report's present the first one in the literature from Colombia.

## 5 Conclusion

In conclusion, ALPPS procedure should be considered as an alternative surgery in patients with liver tumors that don't have enough remnant liver tissue after surgery, with the principal aim to avoid postoperative liver failure; and to achieve R0 resections, with positive oncologic outcomes in terms of DFS; and OS, with acceptable morbidity and mortality rates. However recent studies show the importance of adequate selection of the patient, as the multidisciplinary approach for preoperative and postoperative care is necessary to reach good postoperative outcomes.

## Provenance and peer review

Not commissioned, externally peer-reviewed.

## Consent

Written informed consent was obtained from the patient for publication of this case report and accompanying images. A copy of the written consent is available for review by the Editor-in-Chief of this journal on request.

## Ethical approval

Ethical approval of institutional committee was made previous publication

## Funding

This research did not receive any specific grant from funding agencies in the public, commercial, or not-for-profit sectors.

## Guarantor

Carlos Eduardo Rey Chaves.

## Research registration number

None.

## CRediT authorship contribution statement

CR, DC, JCS: Conception of the idea, manuscript writing, revision, and edition
IR, LT: Manuscript writing, data analysis.

## Declaration of competing interest

Authors do not declare any conflict of interest.
